# Development of the cerebellar white matter is impaired by postnatal hyperoxia and protected by minocycline

**DOI:** 10.1186/2194-7791-1-S1-A6

**Published:** 2014-09-11

**Authors:** Thomas Schmitz, Till Scheuer, Christoph Bührer

**Affiliations:** Neonatology, Charité, Berlin, Germany

Brain injury of preterm infants has widely been ascribed to the cerebrum, but recent studies demonstrate that injury of the cerebellum occurs, too [1]. Causes of cerebellar pathologies in preterm infants and ways of protection are underinvestigated. In general, perinatal infection/inflammation, hypocarbia, and hyperoxia are factors associated with brain damage in preterm infants [1, 2]. We investigate whether oxidative stress induced by postnatal hyperoxia impairs the development of the cerebellar white matter.

We used a neonatal hyperoxia model in rats with 80% oxygen exposure for 24h from P6 to P7, and determined development and maturation of oligodendroglial precursor cells (OPCs) in the cerebellum after recovery in room air until P9, P11, P14, and P30.

Volume of the cerebellum measured by MRI in P30 rats after exposure to hyperoxia was significantly reduced as compared to control litters always kept in room air (hyperoxia = 209 µL, controls = 232 µL; n=8, t-test p<0.05). Myelination measured by MBP expression in immunostainings and Western blots was significantly reduced from P7 to P30. Numbers of apoptotic TUNEL+NG2+ OPCs were higher in hyperoxia rats; OPC proliferation and maturation towards CC1+ stages were significantly reduced. Analysis of Iba1 immunostainings and of inflammatory cytokine expression did not reveal signs of microglial activation after hyperoxia. Oxidative stress after hyperoxia was indicated by increased nitrotyrosin. Treatment with minocycline significantly attenuated toxic effects of hyperoxia.

Postnatal hyperoxia in rats causes critical features of cerebellar pathology seen in preterm infants. A pharmacological approach for prevention seems feasible.
